# Supplementary material for: Are carbon emissions trading and green financial instruments synergistic? -Comprehensive quantitative research based on content analysis
Source: PLoS One. 2024 Mar 7;19(3):e0298601. doi: 10.1371/journal.pone.0298601 (PMC10919601; doi:10.1371/journal.pone.0298601)
Supplement: S1 Data — (ZIP) [file pone.0298601.s001.zip › Data/地级市Z_F工作报告.zip!/░▓╗╒/┬φ░░╔╜╩╨/└┤╫╘╣1⁄2╓┌║┼í╛┬φ┐╦╩2╛▌═°í┐_2011.docx]

# **政府工作报告（2011）**

【字体大小：[大](http://zwgk.mas.gov.cn/openness/detail/content/javascript:doZoom(18)) [中](http://zwgk.mas.gov.cn/openness/detail/content/javascript:doZoom(15)) [小](http://zwgk.mas.gov.cn/openness/detail/content/javascript:doZoom(14))】

政府工作报告

——2011年1月6日在马鞍山市第十四届

人民代表大会第五次会议上

代市长  张晓麟

各位代表：

现在，我代表市人民政府，向大会作工作报告，请予审议，并请市政协委员和其他列席人员提出意见。

一、2010年工作回顾及“十一五”主要成就

刚刚过去的2010年，是我们克服经济危机复苏过程中的各种挑战、经济社会发展取得显著成效的一年。一年来，在省委、省政府和市委的坚强领导下，市政府团结带领全市人民，认真贯彻党的十七大和十七届四中、五中全会精神，全面落实科学发展观，围绕坚持科学发展、走在全省前列的奋斗目标，大力构建“6653”现代产业体系，加快实施“1255”城市发展战略，经济回升向好势头进一步巩固，社会呈现和谐稳定的良好局面，市十四届人大四次会议确定的目标任务全面完成。

预计全市实现地区生产总值760亿元，比上年增长14%；财政收入140亿元，增长14.5%；全社会固定资产投资725亿元，增长32.8%；社会消费品零售总额147亿元，增长19%；城市居民人均可支配收入22900元，增长12.3%；农民人均纯收入8740元，增长10%。全面小康实现程度达97.6%，比上年提升3.4个百分点。我市成为全国外贸百强城市、中国十佳投资创业城市、全国加工贸易梯度转移重点承接地，跻身长三角经济协调会成员城市行列，马鞍山经济开发区成功晋升国家级经济技术开发区。

过去的一年，我们积极抢抓国家扩大内需的机遇，强力实施“项目推进年”活动，经济保持平稳较快发展。全年重点项目完成投资356亿元，其中工业项目114亿元。马鞍山发电厂“上大压小”扩建、格力电器等115个项目开工建设，马钢结构调整、方圆回转支承等项目加快实施，国星生物等52个项目竣工。工业经济运行持续向好，全市规模以上工业增加值450亿元，增长19.2%。马钢实现销售收入688亿元，星马集团销售收入突破100亿元，山鹰公司等60户重点骨干企业产值增势强劲。建筑业总产值达到140亿元，增长18%。全年完成第三产业增加值210亿元。长运物流港竣工投入运营，欧尚超市、沃尔玛新一城等大型商场开业。龙湾水文化主题度假公园开工建设，全市旅游业总收入增长26%。房地产新开工面积295万平方米，竣工面积186万平方米。截至上年底，各银行业金融机构存款余额810亿元，贷款余额553亿元，新增存贷款均超百亿元。完成进出口总值26亿美元，增长59%。外贸内支线班轮正式开通，公用型保税仓库和出口监管仓库投入运营。

过去的一年，我们着力优化产业结构，推动自主创新和节能减排，经济发展方式加快转变。出台落实工业企业升级行动计划，新增规模以上企业144户、销售收入超亿元企业17户，总数分别达753户、110户。制定加快发展服务业实施意见和行动计划，第三产业投资增长32.4%。积极发展现代农业，当涂现代农业示范区加快建设，农业产业化龙头企业规模进一步扩大，农民专业合作社发展到148家。加强自主创新能力建设，华菱公司、矿院被认定为国家级创新型试点企业，马钢车轮商标被认定为中国驰名商标，生物芯片国家工程研究中心分中心组建授牌。马钢、矿院两家省级院士工作站成立，合工大（马鞍山）高新技术研究院挂牌。全市高新技术产业产值突破400亿元，增长15%；六个新兴产业完成产值100亿元，增长46%。加强节能减排和环境保护，依法关停15户高耗能企业，对全社会电力消费进行调控，东部污水处理厂建成运营，慈湖污水处理厂基本完工，污泥处置工程开工建设。马钢被认定为国家首批“两化融合促进节能减排试点示范企业”，全市万元地区生产总值综合能耗下降5.1%，超额完成省政府下达的主要污染物减排任务。

过去的一年，我们抢抓皖江示范区建设机遇，着力打造产业承接平台，对外开放进一步扩大。示范园区起步区路网框架拉开，5万平方米农民安置房竣工交付，优异光伏等12个项目开工建设，新的管理体制初步建立。马鞍山经济技术开发区当年财政收入突破10亿元，增长50%以上，晶威电子等41个项目建成投产，华菱3万辆重卡等33个项目加快建设。慈湖经济开发区实际利用外资居全省省级开发区首位，立白10万吨油脂、金星钛白粉技改等项目加快实施，金桐化工等项目竣工投产。精心承办皖江示范区政策推介暨项目对接会、推进皖江示范区建设座谈会，成功举办装备制造、软件动漫、文化旅游、现代农业等专题招商会，格力电器、东风小康汽车、启耀光电、开米日化等项目签约落户。全市实际利用内资380亿元，增长42%；实际利用外资7.2亿美元，增长20%。新发展私营企业2400户、个体工商户9300户，长江钢铁、鑫港炉料两家企业进入全省民营企业前十强。加强与全国知名非公企业合作发展，118个项目签约，总投资781亿元。与长三角一体化发展深入推进，与南京市达成全面合作框架协议，与宿州市结对合作稳步实施。

过去的一年，我们立足城市空间拓展和功能提升，重点实施一批重大基础设施项目，拉开了大规划、大建设的序幕。长江公路大桥、宁安城际铁路有序推进，东环路原址高速化改造基本建成，马濮公路升级改建路基工程基本完工，宁芜铁路扩能改造工程即将开工。314省道升级改造稳步推进，205国道升级改造前期工作基本完成。太白大道等4条城区主干道综合改造完成，湖东南路、沿江大道北段主体工程完工。谋划编制副城区、中心镇和产业功能区规划，完成新区设计。滨江新区沿江大道南段、采石河西路和九华小区加快建设，秀山新区秀山湖、东部环路、霍里山公园综合整治启动。小城镇建设完成投资28.2亿元，增长63.7%。12个电网项目加快推进，高压线廊整合改造有序实施。改善城乡环境面貌，慈湖河上游综合整治工程竣工，滨湖游园建成开放，东湖公园主体工程完工。开展土地整理和村庄整治，当涂县被列为全省首批推进整村整治示范县。通过第十次土地卫片执法检查。市容管理社会化、环境卫生管理市场化运作试点扎实开展，主干道机械化清扫作业全面推行。

过去的一年，我们深化重点领域改革，推动体制机制创新，经济社会发展活力明显增强。政府机构改革全面完成，事业单位岗位设置管理、绩效工资改革实施。对市直129家行政事业单位产权实施统管。深化医药卫生体制改革，成为公立医院改革国家联系试点市，基层医药卫生体制综合改革扎实推进，实现阶段性医改目标。组建市艺术剧院，在全省率先完成文化体制改革。资源性产品价格改革稳妥推进。马钢产权多元化改革迈出新步伐，与长钢签订合资框架协议。深入推进城乡一体化综合配套改革，新建农村道路65公里，重点农田水利工程加快建设，“农家店”覆盖所有乡镇和90%以上行政村。在全省率先建成覆盖城乡的数字文化服务体系，42家农家书屋建成使用。农村电网完善工程和新农村电气化建设顺利推进。新型农村养老保险参保率提升到80%，新型农村合作医疗参保率达100%。加大金融创新力度，20亿元城市建设债券获准发行，皖江城市带承接产业转移投资基金首期募集7亿元，闽江等融资担保公司揭牌。马鞍山农村商业银行完成增资扩股，发起设立的两家外地村镇银行开业。江阴农村商业银行当涂支行挂牌营业，光大银行、招商银行马鞍山分行加紧筹建。泰尔重工、鼎泰新材、现代牧业成功上市。

过去的一年，县区充分发挥各自优势，全力以赴加快发展，综合实力显著提升。当涂县实现地区生产总值175亿元，增长16%；规模以上工业增加值80亿元，增长32%；财政收入22亿元，增长22%，当涂经济开发区家电、汽车零部件两个产业集群初具雏形。三区固定资产投资均超百亿元，财政收入平均增幅达53.4%。花山区雨山路东段商圈初步形成，软件园一期工程基本完工，被授予“省级服务外包示范区”，吉联国际软件园、长江泛太科技产业园项目签约。雨山工业园获准筹建省级开发区，动漫游戏产业基地签约企业120家，清华紫光等知名企业入驻，国家级（大学生）网络游戏动漫实验园加快推进，国家级再生资源集散市场即将竣工。金家庄区规模以上工业产值突破百亿元，当年搬迁居民3543户，累计7167户、2.2万人。县区按照全市统一部署，在农村征迁、城市房屋拆迁和城乡拆违工作中发挥主体作用，全市累计完成拆迁20573户，拆迁面积340万平方米，交地47310亩，拆除各类违法建设93万平方米，征迁拆迁拆违集中行动取得阶段性成果。

过去的一年，我们着力保障和改善民生，全面发展各项社会事业，社会保持和谐稳定。投入资金14.3亿元，实施50项民生工程，惠及全市城乡居民。实施积极的就业政策，全年缓缴、减免税费及兑现补贴2.45亿元，城镇登记失业率降至3.12%。养老保险关系跨省转移接续工作稳步推进，生育保险制度实现城乡全覆盖，马钢医疗工伤生育保险与市本级完成并轨，城乡低保标准居全省前列。市社会福利服务中心项目加快推进，雨山区被评为“全国养老服务示范单位”。启动老旧小区综合整治三年计划，首批18个小区整治开始实施。33642套安置房开工建设，5979套竣工；4个危旧房改造项目完工，11个城市和国有工矿棚户区改造项目加快推进，廉租住房保障范围和标准位居全省前列。住房公积金制度覆盖面扩大，在支持廉租房建设中发挥了积极作用。出台落实21条稳定市场价格措施，群众基本生活得到保障。社会主义核心价值体系建设经验受到中央和省级主流媒体关注。在全国部分城市公共文明指数测评中获得较好成绩。第九届省艺术节暨马鞍山中国李白诗歌节隆重举办，“城市之光”群众大巡游等文化活动精彩纷呈，《千羽锦》、《姑溪情歌》剧目受到各方好评。大剧院建成投入使用，科技馆、青少年宫、妇女儿童活动中心主体封顶，市体育会展中心开工建设。中小学校舍安全工程全面实施，二十中建成使用，中职园区加快建设，工业学校通过国家示范中等职业学校评估，马鞍山师专二期工程启动建设。人民医院新门诊大楼、中医院南迁一期工程竣工使用，提前完成基层医疗卫生服务机构标准化建设任务。低生育水平持续稳定，第六次全国人口普查全面展开。在全省率先实现体育健身工程全覆盖，学校体育场馆全部向社会开放，我市运动员在第十二届省运会上获得金牌第四、奖牌第五的好成绩。加强社会治安综合治理，强化综治基层基础工作和严打整治专项行动，组织开展为重点工程创平安、技防“五进”、创建“零发案小区”活动，八类严重暴力犯罪案件下降19%，人民群众安全感和满意度进一步提高。世博会和亚运会安保任务顺利完成，校园安保工作全面落实。开展领导开门接访、带案下访及重点信访案件牵头大调解活动，重点信访积案有效化解。突发事件应急预案、处置体系和管理机制进一步健全。医疗纠纷、道路交通事故赔偿第三方调处机制建立。通过“五五”普法考核验收。“安全生产年”活动扎实开展，安全生产形势保持平稳。双拥创建不断深入，优抚对象抚恤补助标准提升到全省首位，国防教育和后备力量建设得到加强。民族团结、宗教和睦局面继续巩固。人防、地方志工作进入全国先进行列，外事、侨务、对台、档案工作继续加强，地震、气象、防灾减灾工作扎实推进。老干部活动中心基本完工，老年大学南校区投入使用，青少年和妇女儿童工作取得新进展，残疾人和红十字事业取得新进步。

过去的一年，在加快经济社会发展的同时，我们更加注重政府自身建设。政府系统创先争优和“三民一促”活动扎实开展，“学习《廉政准则》、规范从政行为、促进科学发展”主题教育活动取得明显成效。市政府认真执行市人大及其常委会决议、决定，定期向市人大常委会报告工作、向市政协通报情况，主动接受市人大的法律监督、工作监督和市政协的民主监督。政府系统承办的5件人大议案、99件建议批评意见、294件政协提案全部办复。在全省率先实施法治政府指标体系考核，创新开展市政府行政复议委员会试点。构建建设项目联合审批服务平台，实现全市行政机关政府信息公开全覆盖，首次开展民主考评百名科长、公共服务行业和市场中介组织专项效能评议活动。加大重点工作督查督办力度，目标管理激励导向作用有效发挥。强化行政监察和审计监督，加强对新增中央投资项目监督检查和工程建设领域突出问题专项治理，严肃查处涉及征迁拆违中的违法违纪行为。建立行政权力规范运行预警机制，启动政府投资重点项目试行廉政风险点管理，推进“阳光村务”工程，惩治和预防腐败体系建设深入推进。

2010年全市经济社会发展取得显著成绩，为“十一五”规划的全面超额完成画上了圆满的句号。

这五年，是经济实力显著增强的五年。全市生产总值由349.5亿元增加到760亿元，年均增长15.5%，人均生产总值超过8600美元；全社会固定资产投资累计2270亿元，是“十五”时期的4.2倍，年均增长30.5%；财政收入由64亿元增加到140亿元，年均增长16.9%；城市居民人均可支配收入由11935元提高到22900元，年均增长13.9%；农民人均纯收入由4511元提高到8740元，年均增长14.1%。

这五年，是结构调整取得突破的五年。工业结构持续优化，马钢新区等一批重大项目相继建成投产，五年新增规模以上工业企业464户，规模以上工业增加值年均增长20%，高新技术产业增加值占规模以上工业增加值的比重达32.5%。服务业总量不断扩大，层次快速提升。传统农业加速向现代农业转变。

这五年，是对外开放持续扩大的五年。累计实际利用外资25亿美元，利用内资超过1100亿元。全市形成1个国家级经济技术开发区、4个省级开发区和一批特色工业园区梯度跟进、错位发展的格局，示范园区建设开局良好。马鞍山口岸实现对外籍轮开放，马鞍山港跻身长江港口十强行列。对外贸易总额累计超过120亿美元。对外友好交往不断扩大。

这五年，是各项改革不断深化的五年。政府机构、行政管理、财税体制、教育体制改革深入推进。马钢、十七冶、矿院、中冶华天等企业改革顺利实施。深化医药卫生体制改革，公立医院改革试点、基层医药卫生体制综合改革快速推进。金融创新迈出重要步伐，一批金融机构相继落户。5家企业成功上市，上市公司增至8家，股票9支，市值接近3000亿元。

这五年，是城乡面貌大为改观的五年。城市建成区面积由66平方公里扩大到78.5平方公里，城镇化率年均提升2个百分点，达到68%。长江公路大桥等重大交通设施项目相继开工建设，新建、改扩建及在建公路总里程720公里。主城区向两翼拓展迈出坚实步伐，副城区集聚辐射功能明显增强，中心镇发展活力进一步激发，城乡一体化发展格局逐步形成。

这五年，是社会建设全面加强的五年。连续4年实施民生工程，累计投入资金超过36亿元。统筹城乡就业、创建创业型城市、城镇居民医疗保险、新型农村养老保险、居民生育保险、“金保工程”建设6项工作被列为全国试点。荣获全国文明城市、国家环保模范城市、全国绿化模范城市、全国双拥模范城市等荣誉，城市知名度和影响力大幅提升。

各位代表，“十一五”成就来之不易，令人鼓舞。这是按照中央和省委、省政府的决策部署，在市委的坚强领导下，在市人大、市政协的监督帮助下，在社会各界的大力支持下，全市各级政府依靠广大人民群众共同奋斗的结果。在此，我代表市人民政府，向辛勤工作在全市各条战线上的广大干部群众，向市人大代表、市政协委员，向各民主党派、工商联、人民团体和社会各界人士，向中央和省驻马单位、来马投资企业，向驻马解放军、武警官兵和公安民警，表示衷心的感谢并致以崇高的敬意！

在肯定成绩的同时，我们也清醒看到，当前还存在一些亟待解决的突出问题，主要是：企业数量偏少、体量不大，牵动性大项目不多，战略性新兴产业、现代服务业尚在培育，加快发展的内生动力有待增强；土地等资源要素供给矛盾日渐突出，资源环境压力持续增大，开发园区亟待扩容升级，中心镇辐射带动力较弱，转型发展压力大；农民收入稳定增长的基础不够牢固，困难群众生产生活条件有待改善；少数机关干部发展紧迫感不强、责任感缺失、紧张感不够，工作节奏慢、服务不到位，一定程度地影响到工作落实和效能提升。对于这些问题，我们将高度重视，采取措施切实加以解决。

二、“十二五”发展的指导思想和目标任务

“十二五”时期是马鞍山推动产业转型升级、实现发展方式转变的攻坚期，是统筹城乡发展、推进城乡一体化的加速期，是建设更高水平小康社会、向基本实现现代化目标迈进的关键期。纵观全局，我们处于大有可为的黄金发展期。国家转变发展方式导向十分鲜明，为我们调整优化经济结构提供了现实机遇；国内外产业转移趋势愈加明显，为我市发挥比较优势、更好地承接产业转移提供了广阔空间；城镇化加速发展带来的巨大需求，为我们经济发展增添了持续动力；创新密集和产业振兴时代的到来，为我们推动科技创新、培育战略性新兴产业提供了有利条件；国家实施扩大内需政策，支持皖江示范区建设，必将为我市发展带来更多的政策支持。我们也要看到，尽管我市人均经济水平较高，但经济规模不大、增长速度不快、综合实力不强仍是我们面临的主要矛盾。特别是当前沿海发达地区在加速转型升级中谋求更高层次跨越，省内兄弟城市呈现出你追我赶的竞相发展态势。标兵越跑越远，追兵已到跟前，已将我们置于不进则退、慢进也是退的紧要关口。我们务必要增强忧患意识，树立新标杆，推动大发展，比人均增强信心，比总量和速度自我加压，勇做皖江城市带的“箭头”，敢在融入长三角中“带头”，以时不我待、争分夺秒、急起直追的胆识和气魄，奋力提升在区域经济格局中的位次。

根据中共马鞍山市委《关于制定国民经济和社会发展第十二个五年规划的建议》，“十二五”时期全市经济社会发展的指导思想是：以邓小平理论和“三个代表”重要思想为指导，深入贯彻落实科学发展观，以科学发展为主题，以加快转变经济发展方式为主线，坚持转型发展、加快发展、和谐发展，实施工业主导、城乡一体、创新推动、开放带动、民生优先、可持续发展战略，全力推动经济社会更高更大更快发展，确保“十二五”持续走在全省前列，在全省率先建成全面小康社会，为率先基本实现现代化奠定坚实基础，努力建设和谐幸福马鞍山。

“十二五”时期全市经济社会发展的主要目标是：

——综合实力跃上新台阶。在坚持提高经济质量的前提下，地区生产总值、财政收入比2010年翻一番以上，主要经济指标增速位于皖江城市带前列，人均值保持全省第一，综合实力居全省前三强。

——结构调整取得新突破。现代产业体系基本形成，战略性新兴产业和现代服务业增加值翻两番。科技创新能力显著提升，企业综合竞争力进一步增强。资源利用效率显著提高，节能减排指标全面完成，生态环境质量明显改善。

——城市发展展现新面貌。城市规模逐步扩大，功能不断完善，综合承载力明显增强，城市化水平不断提高，城乡一体、宜业宜居的现代化大城市框架基本形成。

——改革开放迈出新步伐。市场经济体制更加完善，承接产业转移示范区建设取得显著成效，区域合作进一步深化，开放型经济水平不断提升，经济发展活力明显增强。

——人民生活得到新改善。城乡居民收入翻一番，覆盖城乡居民的基本公共服务体系逐步完善，社会建设显著加强，社会管理水平明显提高，城乡文明程度进一步提升，社会更加和谐稳定。

“十二五”力争目标概括为“12431”：全社会固定资产投资累计超过1万亿元，地区生产总值达到2000亿元，财政收入达到400亿元，综合实力居全省前三强，城乡居民收入翻一番。

“十二五”时期全市经济社会发展的主要任务是：

努力扩大有效需求。坚持向上争取资金、招商引资、激活民资和扩大融资“四资并举”，加快推进项目建设，完善项目管理体制，突破项目建设瓶颈制约。大力拓展消费需求，开拓南京市场，努力扩大品牌影响，提高市场占有率。

加快推进结构优化升级。推动马钢成为全省首个销售收入过千亿元企业，支持华菱公司形成年产10万台重卡生产能力，建成全国重要的装备制造业基地和全国有影响的食品加工基地。加快发展战略性新兴产业，建成高新技术产业基地。积极发展服务业，建成区域性现代服务业集聚区。加快推进农业园区建设，促进农业转型升级。

着力提高城市化水平。提升中心城区功能和品位，推进副城区建设，抓好中心镇建设。加快马鞍山经济技术开发区扩容升级，实现示范园区“五年大发展”目标，快速推进慈湖港口物流加工区建设，高水平开发江心洲生态旅游区，加快建设濮塘休闲度假区。深入推进新农村建设，加速城乡一体化进程。

推动科教兴市和人才强市。推进国家技术创新工程试点工作，争创国家知识产权示范市，力争马鞍山经济技术开发区成为国家级高新技术开发区。完善科技创新体制，健全以企业为主体的产学研合作机制。深化教育改革，加大教育投入。统筹开发人才资源，创新人才体制机制，发挥人才的支撑作用。

不断深化改革开放。推进经济体制改革，优化市场配置功能。继续深化行政管理、医药卫生体制改革，深入推进社会事业体制改革。加大招商力度，创新招商方式，提高利用外资水平。积极参与长三角区域合作，加强与南京、合肥、芜湖、巢湖的对接合作，促进共赢发展。加强与宿州市结对合作。优化出口产品结构，加大新兴市场开拓力度。

加强资源节约和环境保护。严格控制新上高耗能高污染项目，坚决淘汰落后产能，积极发展低碳经济和循环经济。抓好工业污染防治，提高生产生活污水处理能力。完成金家庄部分地区居民搬迁任务。推进生态马鞍山建设，促进城乡生态系统良性循环。

促进文化大发展大繁荣。以社会主义核心价值体系建设为根本，巩固提升全国文明城市创建成果，努力实现全面文明、全体文明、全域文明、全程文明。加快发展文化事业，提高公共文化服务能力，不断丰富群众文化生活。发展文化产业，着力提高文化产业规模化、集约化、专业化水平。

切实保障和改善民生。着力提高城乡居民收入，加大对低收入群体的帮扶救助力度。统筹抓好改善民生和社会事业发展，让群众共享改革发展成果。实施积极的就业政策，加快城乡社会保障一体化建设，不断缩小城乡差距，逐步提高保障水平。加强和创新社会管理，深入推进“平安马鞍山”建设，维护社会大局和谐稳定。

三、扎实做好2011年各项工作  在全省率先建成全面小康社会

2011年是实施“十二五”规划的开局之年，也是我市在全省率先建成全面小康社会的冲刺之年。新的一年，我们要认真贯彻落实中央、全省经济工作会议和市委七届十四次全会精神，主动适应宏观形势的新变化，围绕构建“6653”现代产业体系和实施“1255”城市发展战略，突出抓好事关发展的各项重点工作。综合各种因素，今年经济社会发展的主要预期目标为：地区生产总值增长13%以上；财政收入与地区生产总值同步增长；全社会固定资产投资增长30%以上；社会消费品零售总额增长18%以上；城市居民人均可支配收入、农民人均纯收入增长10%以上；城镇登记失业率控制在4%以内；万元地区生产总值综合能耗下降4%；主要污染物排放量、人口自然增长率控制在省下达指标以内。这是必须完成的目标，实际工作中还要完成得更好些，力争“十二五”有一个良好开局、有一个较高起点。主要抓好以下重点工作：

（一）抓大项目、大企业，着力增强工业发展的内生动力。

**强力实施重点工业项目。**支持马钢加快环保搬迁和本部配套、高速车轮用钢等项目建设，力争完成投资100亿元。推进长钢300万吨精品钢和配套工程建设，完成投资30亿元。加快华菱3万辆重卡实施步伐，确保年底前竣工投产。开工建设格力2000万台小家电、东风小康10万辆微型车、西安开米40万吨洗涤剂项目，推进科达洁能、中钢天源电子材料产业园项目建设，力争马鞍山发电厂扩建、方圆回转支承重型装备、金星钛白粉项目早日竣工投产。积极推进大唐二期等重大项目前期工作，精心谋划一批新的产业项目，创造条件尽快组织实施。

**推动工业企业转型升级。**支持马钢产品开发与创新，提高优势产品比重，加强矿山资源开发，培育非钢支柱产业，力争销售收入突破800亿元。加快山鹰80万吨纸技改、统力风电用回转支承项目建设进度，推进华菱新一代节能环保大功率发动机项目前期工作。发挥大企业和骨干企业带动作用，拓展延伸上下游产业链，形成一批具有较高水平和影响力的中小企业产业集群。鼓励建筑业企业拓展外部市场，推动建筑业快速发展。密切关注市场变化和工业经济走势，加强对重点行业、重点企业的跟踪监测，及时发现苗头性、倾向性问题并向企业预警。全年完成工业投资420亿元，增长20%以上；新增销售收入超亿元企业20户；规模以上工业增加值增长18%以上。

（二）抓新兴产业、服务业，着力加快经济发展方式转变。

**培育发展战略性新兴产业。**集中政策、资金、要素优先支持新兴产业发展，引导金融机构加大对新兴产业的资金投放力度，加快推进优异光伏、天翊光能、启耀光电等项目建设，力争在高端装备制造、新材料、节能环保、电子信息和新能源产业上取得突破。以马鞍山软件园、动漫游戏基地为载体，引进知名企业入驻，培育马鞍山创意产业基地，打造国家级动漫游戏产业基地。

**促进服务业加快发展。**加大城市核心商圈培育力度，加快团结广场核心商务区、雨山路现代商务中心建设，促进特色商业街区发展，引导做大本地消费。加快会展中心建设，积极发展会展经济。重新审视完善商业规划和旅游规划，迎接大桥和城铁时代的到来。加强流通体系建设，兴建和改造一批农产品和生产资料大型专业批发市场，鼓励重点流通企业连锁网络向新区延伸。合理布局和建设商业地产，促进房地产市场健康发展。推动采石风景区创建5A级旅游区，加快濮塘休闲度假区规划编制和招商步伐，促进特色旅游业快速发展。

**增强自主创新能力。**深入实施科技倍增计划，突破重点产业和优势产业领域的重大关键技术，加快产业技术创新步伐，提升主导产业核心竞争力。全年新增高新技术企业15家，高新技术产品30个。支持企业研发机构和平台建设，培育壮大一批具有自主知识产权的科技型中小企业。创新产学研合作模式，加快合工大（马鞍山）高新技术研究院建设，争取吸引更多的高校院所在我市设立研发机构，积极筹建院士工作站。

**推动节能减排。**实施钢铁、化工、发电、矿山等行业的电机改造、余热余压利用、工业炉窑改造、清洁生产等节能改造项目，加快推广成熟实用的节能新技术、新工艺和新材料，实施路灯节能改造。推进饮用水保护工程，加强大气污染防治，基本建成危险废物集中处置中心，加强污染源监控系统建设。确保完成省政府下达的主要污染物排放总量削减指标，顺利通过国家环保模范城市复查。

（三）抓外延拓展、内涵提升，着力增强城市综合承载功能。

**加快重大交通基础设施建设。**推进马鞍山长江公路大桥及连接线建设。积极推进宁安城际铁路建设，启动客运中心枢纽站及站前广场配套工程。完成东环路高速化改造高架路段建设，确保整体工程全面竣工。启动围乌路升级改造及北延工程，加快314省道、205国道升级改造步伐，确保马濮公路升级改建工程竣工通车。推进人头矶港区一期工程，启动建设太平府港口码头。积极筹划城市轨道交通工程，推动姑溪河航道升级、太平府航道开通、扁担河综合整治和石臼湖保护开发利用前期工作。

**力争新区建设出形象。**坚持大规划、紧凑式开发，找准切入点，有序推进新区建设。滨江新区要建成沿江大道南段、采石河路西延工程和九华小区，启动采石古镇整体改造、滨江湿地生态公园建设，认真规划建设“小九华”。秀山新区要突出社会事业优先，加快实施星光大道、向霍路等路网建设，推进秀山湖、东部环路、霍里山公园等重点工程，为新区积聚人气创造有利条件。加快博望新区总体规划编制，统筹推进建设与招商工作。

**完善城乡基础设施和公共配套。**完成采石河路市政化改造、沿江大道北段工程建设，推进江东大道、慈湖河路及霍里山大道南延工程。实施湖西南路、花山路综合改造，续建丰收路、勤俭路、九华路二期。新建花雨广场、解放路等地下停车场，努力解决中心城区“停车难”问题。加快川气东送门站及相关配套设施建设，推动电网改造工程有序实施。完善东部污水处理厂、慈湖污水处理厂管网配套，继续推进慈湖河综合整治工程。完善老城区基础设施，完成39个老旧小区整治任务，进一步规范强化小区物业管理。综合整治城市主出入口，续建南湖公园及花雨广场景观提升工程，新建花果山公园及东郊动植物园。加强中心镇建设，完善产业功能配套，优化中心镇发展环境。

**强化城乡规划管理。**完善规划运行机制和规则，增强规划的科学性和可行性。立足全域马鞍山，对市县基础设施、公共服务设施、产业功能区统一规划布局，推动人口向城镇集中、产业向园区集聚。本着同城化的原则，加强与南京、芜湖的城市规划对接，加快基础设施一体化推进步伐。开展江心洲概念规划招标，提升副城区、中心镇规划水平。构建数字化城市管理平台，深化相对集中行政处罚权工作，推进城市网格化管理，进一步提高综合管理水平。

（四）抓示范园区、开发园区，着力构建产业承接大平台。

**全力推进示范园区建设。**推动起步区基础设施建设，实现路、水、电、气等配套设施全覆盖。加快建设新能源产业园、现代机械装备产业园、现代物流园和电子信息产业园，积极探索共建园区开发新模式。开工建设美格尔电器等项目，快速推进闽江物流园、广大工业园等在建项目，力争早日竣工投产。加快公租房、安置房及商业配套建设，提升产业和人口集聚水平。

**促进开发园区扩容升级。**加强马鞍山经济技术开发区南区基础设施建设，拆迁房屋16万平方米，建成安置房16万平方米以上，建成道路12公里，实施华菱汽车技术中心、蒙牛四期等项目。推动慈湖经济开发区扩区，加快港口物流加工区、台湾工业园和华福、优科、中海项目建设。推动当涂经济开发区北区产业转型升级，实现南区与太白工业集中区组团发展。促进花山经济开发区提档升级，推动雨山经济开发区电子材料产业园建设，以博望工业园为龙头创建省级经济开发区、国家特色产业园。

**着力提升招商有效性。**创新招商方式，结合产业发展现状，围绕延伸优势产业链，有针对性地开展产业招商和专业招商。加强招商引资工作市级统筹，构建招商信息筛选、项目评估、政策把关、落地开工机制，确保项目招得来、落得下，早开工、早见效。完善绩效考核评价办法，建立分类指导、分类考核机制，最大限度地激发招商引资动力和活力。强化招商引资服务，再造招商工作流程，实施项目全程代理。全年引进外资8.3亿美元，引进内资500亿元以上。提升对外经贸合作层次和水平，全年外贸进出口总额增长15%以上。

（五）抓用地保障、资金来源，着力破解制约发展的瓶颈。

**全面加强土地利用管理。**建立有序顺畅的土地管理机制，健全集体决策、净地出让、双向约束、用地规模预审、建设项目开竣工清理等制度，切实做到依法保护、节约集约、保障供应。改革土地指标分配方式，按项目统一规划、分期供地，实现土地资源效益最大化。加强土地利用管理知识培训，理清土地管理工作流程，提高各级干部土地利用管理水平。扎实开展闲置土地清理、土地指标置换、违法用地查处专项行动，做好第十一次土地卫片执法迎检工作。进一步完善征迁政策，深入推进征迁拆迁拆违行动，全年完成房屋拆迁260万平方米，交地2万亩。坚持疏堵结合，进一步细化完善农民建房审批政策，加强违法建设的巡查和拆除，建立健全城乡高效快捷拆违控违机制。

**千方百计拓宽融资渠道。**推进投融资管理体制改革，实行决策、管理、执行、监督相分离。加强工程建设管理，逐步实行规划、建设、立项、招标、投资、施工“六分开”。对所有工程、信息化等政府投资项目，推行项目统建、资金统管、开工审批表“两统一表”制度。不断丰富金融体系，推动招商银行、光大银行、民生银行马鞍山分行早日开业运营，至少再引进3家银行，新设立20家小额贷款公司、15家融资性担保公司、2家典当行、2家证券营业部。引导金融机构创新金融产品，稳定实质性贷款增长态势。加强对金融机构的考核激励，支持金融业发展壮大。积极推进惊天液压、华骐环保、奥盛新材料、华菱西厨上市进程，新增上市公司3家。

（六）抓城乡统筹、农民增收，着力加快城乡一体化进程。

**加快发展现代农业。**推进当涂现代农业示范区建设，努力争创国家级现代农业示范区。推动农产品加工集中区建设，引导一批龙头企业向园区集中，提升农业产业化水平。培育新型农业服务组织，加快农民专业合作社发展。健全农村土地流转市场化机制，促进农村土地加速流转。完善现代农业区域布局，组织实施当涂县新增1.65亿斤粮食生产能力规划，创建生态休闲旅游农业示范点。抓好农业科技成果转化和应用，提高农业科技创新和推广能力。

**改善农村生产生活条件。**综合实施农村公路提升和大型排涝站更新改造等水利工程，进一步提升农村电网供电保障能力，完成江心洲洲头保护工程。推动农村清洁工程，实施“绿色马鞍山”建设行动，做好全国绿化模范城市复查迎检工作。强化农村安全饮水工程管理，保障饮水质量。健全农村公共文化服务网络，加强公共文化设施管理，深入实施农民体育健身工程。完善农村商品流通网络，改善农村消费环境，挖掘农村消费潜力。

**多渠道促进农民增收。**继续做好农民就业创业工作，逐步建立农民收入内生增长长效机制。加大农民工外出务工指导和服务力度，加强农民工技能培训和新型农民培训，引导农村劳动力多途径就近就地就业和平稳有序转移。积极发展农民创业园，优化返乡农民工创业环境。大力发展休闲观光农业和乡村旅游业，拓展农民增收新空间。努力培育新型农民和产业工人，加快农民市民化步伐。

（七）抓重点领域、关键环节，着力把各项改革引向深入。

**深化社会事业改革。**推进公立医院改革试点，建立公立医院与基层医疗卫生分工协作机制。巩固基层医药卫生体制综合改革成果，实现基本药物制度全覆盖。深入推进基本公共卫生服务均等化，逐步扩大基本公共卫生服务项目范围。深化完善文化体制改革，推进公益性文化单位劳动分配和社会保障制度改革。按照政府花钱买服务的方向，推动社会事业经费供给方式改革。

**推进其他领域改革。**深化行政管理体制改革，着力转变政府职能。推进行政审批制度改革，探索网上预审工作机制。深化招标采购监管体制改革，实行产权出让最高价者得制度，探索建立建设工程有效最低价中标制度，健全完善集中统一规范的招标采购交易市场。完善政府预算体系，改革项目支出预算编制办法，提高财政科学化精细化管理水平。稳步推进事业单位分类改革，深化事业单位人事管理和工资收入分配制度改革。推进城乡一体化综合配套改革，扩大县区经济社会管理权限，充分调动中心镇加快发展的积极性。

**大力发展非公经济。**进一步放宽市场准入，营造多种所有制经济公平竞争的环境。鼓励非公企业开发拥有自主知识产权的主导产品、关键技术，提高核心竞争力。加大对中小企业信贷支持力度，建立面向中小企业的金融服务体系和信用担保体系，为中小企业和金融机构搭建合作平台。引导有创业意愿、创业条件的城乡各类人员进入创业园兴业，激发全民创业活力。

（八）抓文化发展、公共服务，着力推动社会全面进步。

**增强城市文化软实力。**加快公共文化服务体系建设，建成市科技馆、青少年活动中心和妇女儿童活动中心并运行，加快市体育会展中心一期工程建设步伐。发展文化产业，加大济美坊文化产业基地、影视休闲基地建设，做好李白文化博览园前期工作。加强文化遗产保护，加大文艺精品创作。继续办好李白诗歌节。开展群众体育运动，建设全民健身活动中心，举办全市第十届运动会。

**巩固提升文明创建成果。**统筹城乡精神文明建设协调发展，拓展群众性精神文明创建活动，健全文明创建任务目标考核和责任追究制度，确保通过全国文明城市复评。以社会主义核心价值体系建设为导向，深化系列主题教育活动，广泛开展志愿服务。不断净化有利于未成年人健康成长的社会文化环境，争创第三批全国未成年人思想道德建设工作先进城市。

**坚持教育优先发展战略。**重视发展学前教育，做到统一规划、统一布局、统一建设，着力构建学前教育公共服务体系。推进教育均等化发展，逐步让农村学校与城市学校一样享受优质的教育资源，努力让全市的孩子都能“上好学”。基本完成中小学校舍安全工程，启动公办初中加强发展工程，加快南部高中建设，完成中职园一期工程。推进安工大东校区、文天学院二期、职业技术学院二期建设，促进高等教育加快发展。

**完善医疗卫生服务体系。**加快推进市人民医院外科综合大楼、妇幼保健院住院楼改扩建及中医院二期工程建设，着力改善城乡居民就诊住院环境和条件。制定实施区域卫生、医疗机构和公立医院设置发展规划，合理配置医疗资源。启动健康城市建设，确保通过国家卫生城市复审考核。完成第六次全国人口普查工作。健全计生利益导向机制，完善计生服务体系，稳定适度低生育水平。

（九）抓就业、社会保障，着力保障和改善民生。

**深入实施民生工程。**进一步加大民生工程投入力度，着力解决群众最关心、最直接、最现实的生产生活问题。在有线数字电视1项工程完成和统筹城乡就业体系建设、完善农民工工资支付、健全城镇老年居民养老保险、建立农民生产发展互助资金组织等4项工程纳入常态化管理的基础上，实施45项民生工程，投入资金17.3亿元，增长21%。全面加快安居工程建设，新开工安置房11978套、廉租房3000套，建成安置房30000套、廉租房2028套，力争让过渡期满18个月的所有征迁拆迁户在2012年春节前搬入新居。抓好“菜篮子”产品生产和市场供应，加强对重要商品供求价格信息发布，做好稳定市场价格工作。深入实施食品药品放心工程，确保人民群众饮食用药安全。

**加强就业和社会保障。**加快乡镇劳动保障工作平台标准化建设，强化城乡困难人员的就业援助，分类开展就业技能和创业培训。全年新增城镇就业2.54万人，其中下岗失业人员再就业1万人，新增农村劳动力转移就业1.2万人。完善城乡居民养老、医疗保险制度，确保城镇职工养老失业医疗保险参保率保持在98%以上，力争城乡居民生育保险参保率达90%以上，新型农村养老保险参保率达80%以上。认真做好马钢医疗工伤生育保险与市本级并轨运行工作。

**维护社会和谐稳定。**深入推进社会矛盾化解，加快数字化社会治安防控体系建设，改革创新警务机制，进一步提升维护社会稳定的能力和水平。加强信访综合治理，建立县区、乡镇（街道）、村（社区）三级矛盾纠纷调处平台，畅通信访渠道，满足群众合理诉求。启动“六五”普法工作，加强特殊人群服务管理。深入开展特色社区创建活动，完成第八届村民委员会换届选举。强化安全监管，严格落实安全生产责任，深入开展隐患排查治理。巩固民族团结进步事业成果，促进民族关系、宗教关系和谐。高度重视发展老龄事业，切实保障妇女、儿童和未成年人合法权益，逐步健全残疾人服务保障体系。积极推进国防动员、后备力量建设和军民融合式发展，全力做好新一轮全国双拥模范城创建工作。

（十）抓执行力、公信力，着力建设人民满意政府。

新机遇、新挑战对各级政府提出了新的更高要求，必须加强行政能力建设，推进政府管理创新，增强服务意识，努力建设负责、高效、务实政府。

**要高度负责。**在一定程度上，责任心比能力更重要。政府系统工作人员要强化发展意识、机遇意识和危机意识，增强时不我待、只争朝夕、急起直追的紧迫感和食不甘味、寝不安席、坐立不安的责任感，始终保持昂扬向上、奋力赶超、争创一流的精气神。要认真钻研本行业务，把工作搞清楚，把问题搞明白，真正成为行家里手。要在其位尽其责，不说“不行”，要说“怎样才行”，少找干不成的理由，多想干成事的办法。要坚持发展为上、干成为算，敢于碰硬，勇于负责。加大治庸罚懒力度，严格行政问责，努力形成全市上下时时在状态、人人有激情、事事争先进的氛围。

**要务实高效。**为民要真心、工作要真干。要坚持察实情，说实话，办实事，求实效，静下心来谋工作，扑下身子抓落实，多干当前最紧迫的事，多干有利于发展的事，多干群众受益的事。要保持雷厉风行、真抓实干的务实作风，摒弃浅尝辄止、不深入研究的肤浅做法，善于深思，做到极致。创新政府工作流程，推行扁平化管理，减少中间环节，提高工作效率。精简会议和文件，减少事务性应酬，把更多的精力用在谋事干事上。改进机关工作作风，整治不作为、慢作为和乱作为。坚持按法律办事，按制度办事，按规矩办事，主动接受人大及其常委会的法律监督和政协的民主监督，健全完善政府工作规则，深入推进法治政府建设。

**要快速落实。**政府每项工作都必须实打实地做、快节奏地干、严要求地抓。要把复杂的任务具体化、项目化，逐一分解到岗，“点将”到人。工作一经安排布置，要紧盯不放、一抓到底，事事有着落，件件有回音。要大力弘扬“五加二”、“白加黑”的奉献精神，主动排难而进，快速抓好落实，努力将每一项工作做到完成任务、不出问题、群众满意的效果。要严格目标管理，加强督查督办，严查不落实的事，处理不落实的人，解决不落实的问题，确保政令畅通。严格落实党风廉政建设责任制，健全完善惩治和预防腐败体系，规范重要领域权力运行，提高行政决策运行的透明度，加强行政监督和审计监督，从源头上防治腐败，以实际行动树立政府勤政廉洁的良好形象。

各位代表，我们已经迈上了转型发展、加快发展、和谐发展的新征程，前景无限美好，责任催人奋进。让我们高举中国特色社会主义伟大旗帜，在省委、省政府和市委的正确领导下，紧紧依靠全市人民，开拓创新，奋力拼搏，在全省率先建成全面小康社会，为开创科学发展新局面、建设和谐幸福马鞍山而努力奋斗！
